# Curcumin and Chronic Kidney Disease (CKD): Major Mode of Action through Stimulating Endogenous Intestinal Alkaline Phosphatase

**DOI:** 10.3390/molecules191220139

**Published:** 2014-12-02

**Authors:** Siddhartha S. Ghosh, Todd W. B. Gehr, Shobha Ghosh

**Affiliations:** 1 Division of Nephrology, Department of Internal Medicine, Virginia Commonwealth University Medical Center, Richmond, VA 23298, USA; E-Mails: ssghosh@vcu.edu (S.S.G.); tgehr@mcvh-vcu.edu (T.W.B.G.); 2 Division of Pulmonary and Critical Care, Department of Internal Medicine, Virginia Commonwealth University Medical Center, Richmond, VA 23298, USA

**Keywords:** inflammation, cytokines, atherosclerosis, diabetes, intestinal permeability, intestinal alkaline phosphatase

## Abstract

Curcumin, an active ingredient in the traditional herbal remedy and dietary spice turmeric (*Curcuma longa*), has significant anti-inflammatory properties. Chronic kidney disease (CKD), an inflammatory disease, can lead to end stage renal disease resulting in dialysis and transplant. Furthermore, it is frequently associated with other inflammatory disease such as diabetes and cardiovascular disorders. This review will focus on the clinically relevant inflammatory molecules that play a role in CKD and associated diseases. Various enzymes, transcription factors, growth factors modulate production and action of inflammatory molecules; curcumin can blunt the generation and action of these inflammatory molecules and ameliorate CKD as well as associated inflammatory disorders. Recent studies have shown that increased intestinal permeability results in the leakage of pro-inflammatory molecules (cytokines and lipopolysaccharides) from gut into the circulation in diseases such as CKD, diabetes and atherosclerosis. This change in intestinal permeability is due to decreased expression of tight junction proteins and intestinal alkaline phosphatase (IAP). Curcumin increases the expression of IAP and tight junction proteins and corrects gut permeability. This action reduces the levels of circulatory inflammatory biomolecules. This effect of curcumin on intestine can explain why, despite poor bioavailability, curcumin has potential anti-inflammatory effects *in vivo* and beneficial effects on CKD.

## 1. Introduction

**Figure 1:**
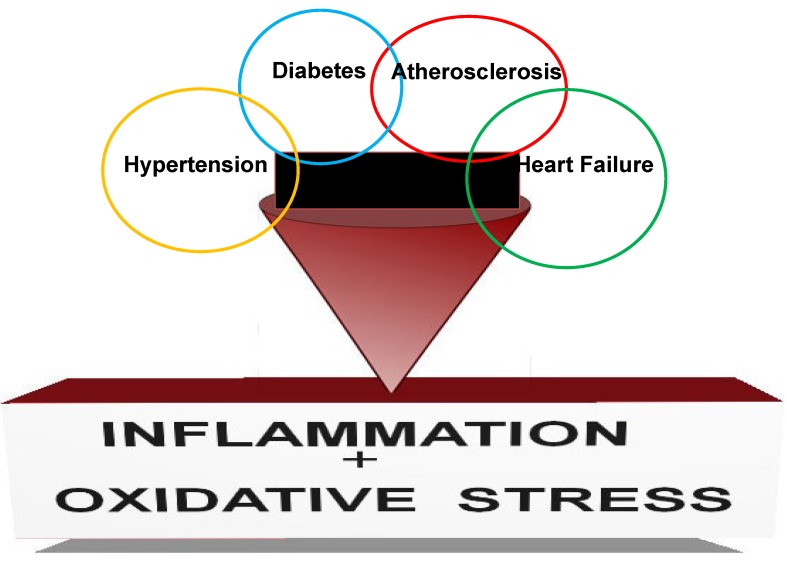
Hypertension, diabetes and atherosclerosis are the major disease which can lead to chronic kidney disease (CKD). Cardiovascular disease such as heart failure is the major cause of death in patients with CKD. However, CKD can also worsen prognosis of hypertension, diabetes, atherosclerosis and heart failure. Inappropriate inflammation and tolerance of oxidative stress plays a significant role in the development and prognosis of these disease processes.

Chronic kidney disease (CKD) is characterized by progressive loss of kidney function which decreases the ability of the body to eliminate soluble waste resulting in the accumulation of “uremic toxins” [1,2]. It is now well documented that CKD is an inflammatory disorder and uremic toxins play a major role in creating the inflammatory milieu [1,2]. CKD is defined by either a reduction in glomerular filtration rate (GFR) and/or the presence of abnormalities in the urine such as protein, red blood cells or white blood cells. The GFR is mathematically derived from serum creatinine and is used to classify the severity of CKD into five stages (Table 1). CKD was ranked 27th in the list of causes of total number of global deaths in 1990 by the Global Burden of Disease Study, but rose to 18th in 2010 [3]. This degree of movement up the list was second only to that for HIV and AIDS. The overall increase in years of life lost due to premature mortality was third largest, behind HIV/AIDS and diabetes mellitus [4]. Although CKD often leads to the development of end stage renal disease (ESRD) it is more importantly an independent risk factor for the development of cardiovascular disease (CVD). Indeed, the most common cause of death in patients with CKD relates to this high incidence of CVD. The high risk of CVD in CKD patients is attributable to a number of other risk factors that accompany CKD such as diabetes mellitus, hypertension, obesity, tobacco abuse and dyslipidemia [5]. An underlying abnormality that unites all of these disorders is the presence of inflammation and oxidative stress. Figure 1 depicts this relationship and highlights the interrelationships between these disorders. Therefore, therapeutic approaches which inhibit inappropriate inflammation and tolerance of oxidative stress have a potential therapeutic value in ameliorating CKD.

**Table 1:** Stages of chronic kidney disease.

Stage	Description	GFR (glomerular filtration rate) mL/min/1.73 m^2^
1	Kidney damage with normal or increased GFR	≥90
2	Kidney damage with mild decreased GFR	60–89
3	Moderate decreased GFR	30–59
4	Severe decreased GFR	15–29
5	Kidney Failure	<15 (or dialysis)

Chronic kidney disease is defined as either kidney damage or GFR <60 mL/min/1.73 m^2^ for ≥3 months. Kidney damage is defined as pathologic abnormalities or markers of damage, including abnormalities in blood or urine tests or imaging studies.

Curcumin is the active ingredient in the traditional herbal remedy and dietary spice turmeric (*Curcuma longa*). It is undergoing clinical trials for various diseases such as cancer, Alzheimer’s disease, ulcerative colitis, *etc.* [6,7]. There are several reviews showing that curcumin can modulate enzymes, cytokines, transcription factors, growth factors, receptors, micro RNA (miRNA), signaling molecules and reactive oxygen species and thereby positively affect inflammation and oxidative stress [7,8,9,10,11]. This review will concentrate on the clinically relevant inflammatory molecules playing a role in CKD and explain the mechanisms by which curcumin blunts the generation or action of these inflammatory molecules and abates CKD.

## 2. Inflammatory Mediators Playing a Role in CKD

### 2.1. Eicosanoids

Experimental and clinical data highlight the role of eicosanoids in CKD as mediators of renal failure progression or as markers of systemic oxidative stress and inflammation. Cyclooxygenase (COX) dependent prostaglandins are associated with inflammation, maintenance of sodium and water homeostasis, control of renin release, renal vasodilation, vasoconstriction attenuation, and prenatal renal development [12]. Intrarenal angiotensin II (Ang II) production, increased sodium delivery, glomerular hypertension, and renal tubular inflammation have been suggested to be responsible for the increase in COX-2 expression [12]. In the rat remnant nephrectomy model, COX-2 is up-regulated after renal fibrosis [13], with a significant negative correlation with renal function. In our study we found that curcumin significantly blocked the expression of renal COX 2 in the kidneys of CKD rats and improved CKD [14]. 5-lipoxygenase (5-LO) another enzyme in the branch of the arachidonate cascade is known to increase the production of 5-hydroxyeicosatetraenoic acid and oxidative stress. 5-LO is profoundly activated in peripheral mononuclear blood cells of CKD patients and in those on dialysis therapy [15]. Mouse genetic studies suggested a functional role for 5-LO in atherosclerosis and 5-LO is also present in human atherosclerotic vessel wall [16]. Curcumin has been known to block 5-LO [9], and the activity of 5-LO isolated from human polymorphonuclear leukocytes was significantly inhibited by curcumin [10]. It is noteworthy, that curcumin blocks phospholipase A2 the key regulatory enzyme in COX and lipoxygenase pathway and ameliorates CKD [14].

### 2.2. Cytokines

Pro-inflammatory cytokines can increase monocyte adhesion, endothelial dysfunction, smooth muscle cell proliferation, oxidative stress, vascular calcification and accelerate atherosclerosis, cachexia and CKD [17,18]. There are large number of pro-inflammatory cytokines but the most the important ones which affect CKD are TNFα, IL-1β and IL-6 [18]. In animal models of CKD we have shown that curcumin significantly reduces the effects of inflammatory cytokines and ameliorates renal injury [14]. In humans early-stage CKD is commonly asymptomatic thus delaying treatment until later symptomatic stages of the disease [19]. Therefore, to better mimic this clinical scenario we initiated curcumin and enalapril (Angiotensin converting enzyme (ACE) inhibitor, routinely used in the treatment of CKD) therapy 6 weeks after the onset of proteinuria, a commonly employed marker of CKD [14]. We demonstrated that curcumin was as effective as enalapril in reducing the inflammatory cytokines TNFα and IL-1β and effectively abated both proteinuria and kidney injury as manifested by glomerulosclerosis and tubulointerstitial injury [14]. In CKD patients marked elevation of IL-6, IL-1β, TNFα and C-reactive protein was observed in the peripheral blood mononuclear cells (PBMC) [20]. When the PBMCs were cultured in the presence of curcumin a significant reduction in the cytokines was noted [20]. In a small clinical study of 16 CKD patients curcumin (824 mg purified turmeric extract, 95% curcuminoids) was given twice daily for 8 weeks [21]. Although creatinine and blood urea nitrogen (BUN) biomarkers for CKD) did not significantly change there was a marked reduction of inflammatory markers TNFα, IL-6 and C-reactive protein. It is noteworthy that these patients also had significant reduction of their BMI [21].

### 2.3. Reactive Oxygen Species (ROS)

During inflammation, cells of the immune system such as macrophages and leucocytes are recruited to the site of the injured kidney. This results in a “respiratory burst” which leads to an overproduction of ROS culminating in oxidative stress. The generated ROS further inflame cells in the kidney leading to the production of soluble mediators such as cytokines, chemokines, and metabolites of arachidonic acid (*i.e*., eicosanoids), which enhances the recruitment of macrophages causing an inflammatory chain reaction in the kidney leading to further damage. In a recent review Choi *et al.* have elaborated various factors which can contribute to the generation of ROS in CKD [22]. NADPH oxidase, xanthine oxidase, and lipoxygenases which initiate ROS production are up-regulated in CKD. Curcumin improves diabetic nephropathy by blunting NADPH oxidase expression [23]. Because the serum uric acid, an important source of oxidative stress in CKD, is produced by xanthine oxidase, blockade of xanthine oxidase by febuxostat led to renoprotective effects in 5/6 nephrectomized rats (animal model for CKD) [24]. In our CKD model we found that curcumin blocked xanthine oxidase expression and significantly improved renal function [25]. Oxidative stress is prevalent in CKD patients and is considered to be an important pathogenic mechanism. The majority of studies investigating anti-oxidant treatments in CKD patients show a reduction in oxidative stress and many show improved renal function [26].

### 2.4. Growth Factors

Growth factors such as TGFβ, VEGF and PDGF play a prominent role in glomerular cell proliferation and glomerular extracellular matrix expansion both of which contribute to renal failure [27]. TGF-β has been shown to mediate several key tubular pathological events during CKD progression, namely fibroblast proliferation, epithelial to mesenchymal transition, tubular and fibroblast ECM production and epithelial cell death leading to tubular cell deletion and interstitial fibrosis [28]. In a randomized, double-blind and placebo-controlled study carried out on 40 patients with overt type 2 diabetic nephropathy a significant drop in plasma TGFβ was observed with curcumin treatment [29]. PDGF (platelet derived growth factor) is known to cause mesangial cell proliferation [30] which appears to play a direct role in the degree of progressive renal injury. VEGF (vascular endothelial growth factor) plays a protective role on a variety of non-diabetic renal diseases however, in diabetes VEGF exhibits deleterious effects on mediating the development/progression of diabetic nephropathy VEGF [31]. Zhang *et al.* have shown that increased VEGF can cause liver fibrosis by disrupting PDGF-β receptor (PDGF-β)/ERK and mTOR [32]. Cardiac hypertrophy following renal failure can lead to heart failure and we have seen that curcumin abolishes the cardiac hypertrophy in CKD animals by disrupting ERK/mTOR pathway [33].

### 2.5. Transcription Factors

Reactive oxygen species (ROS) promotes the expression of pro-inflammatory cytokines and adhesion molecules, which are major mediators of inflammation. Pro-inflammatory TNFα has been known to down-regulate PPARγ, a transcription factor that when activated can have significant anti-inflammatory effects [34,35]. We have shown that PPARγ is down-regulated in rats with CKD, curcumin not only up-regulated PPARγ expression but also abrogated TNFα mediated down regulation of PPARγ [35]. ROS plays an active role in the regulation of transcription factor nuclear factor-κB (NF-κB). In the classical pathway activation of NF-κB is kept in check by IκB which in turn is regulated by an inhibitor of kB kinase complex (IKK). The IKK/NF-κB pathway is a key player in the induction and maintenance of the chronic state of inflammation [8,36,37]. NF-kB and ROS regulate the transcription of a large number of inflammatory cytokines and, in patients with CKD this is associated with vascular dysfunction, inflammation and atherosclerosis [1,38,39]. Furthermore, NF-kB activation leads to the production of several pro-inflammatory cytokines such as TNFα, IL-1β, and IL6, which can further induce ROS formation creating a dangerous cycle between oxidative stress and overproduction of pro-inflammatory cytokines [39]. Aggarwal and colleagues were first to demonstrate the efficacy of curcumin in antagonizing the activation of NF-κB [8]. In the nephrectomized rat model for CKD severe and progressive renal injury was associated with decreased IκB and increased activity of NF-κB [35,40]. TNFα which is known to be responsible for degradation of IκB and activation of the NF-κB system is significantly increased in CKD [34,35,38]. Curcumin treatment decreased TNFα, activation of the NF-κB system, ameliorated renal structural damage, improved proteinuria and creatinine [35].

Whereas, NF-κB plays an important role in the coordinated expression of inflammatory genes, the nuclear factor-erythroid-2-related factor 2 (Nrf2) is the transcription factor that is responsible for both constitutive and inducible expression of antioxidant response element (ARE)-regulated genes. Thus, Nrf2 can regulate antioxidant and anti-inflammatory cellular responses of this system, playing an important protective role on the development of renal failure [41,42,43]. NRF2 activity is primarily governed by the Kelch-like ECH-associated protein 1 (KEAP1). Under normal conditions, NRF2 is continuously degraded by the KEAP1-Cul3-proteasome axis. When Cys residues of KEAP1 protein are modified, conformational KEAP1 changes lead to Nrf2 liberation and transcriptional activation of an array of ARE-bearing genes [22]. Certain molecules such as curcumin, resveratrol, cinnamic aldehyde, and quercetin possess an electrophilic α, β-unsaturated carbonyl moiety called a Michael acceptor. This moiety is highly reactive to the Cys residues of KEAP1, which in turn induces conformational changes of KEAP1 thereby inducing Nrf2 [44,45]. In the remnant kidney tissue of rat CKD model Nrf2 activity (nuclear translocation) was mildly reduced at 6 weeks and markedly reduced at 12 weeks, whereas the Nrf2 repressor Keap1 was up-regulated and the products of Nrf2 target genes were significantly diminished at 12 weeks [42]. These results were confirmed by Soetikno *et al.* when they showed reduced Nrf2 protein expression, and up regulated KEAP1 in the remnant kidney of CKD animals which was reversed by curcumin [46]. Additionally they also showed higher kidney malondialdehyde concentration and lower glutathione peroxidase activity, which was associated with the up-regulation of NADPH oxidase subunit, p67phox and p22phox, NFκB, p65, TNFα, TGFβ, cyclooxygenase-2, and fibronectin accumulation. All of these changes were corrected by curcumin [46].

### 2.6. Immune System

CKD induces several changes in the innate and adaptive immune systems and, collectively, they may have a strong influence on increased atherogenesis and vascular disease seen in CKD. Progressive CKD is associated with deleterious effects on the innate immune system, such as decreased phagocytic ability and depletion and dysfunction of denditric cells. CKD plays a key role in macrophage migration, increased endothelial trapping of macrophages and decreased macrophage egress from the artery [47]. Furthermore, increased macrophage infiltration into the kidney and other tissues contributes to the release of pro inflammatory cytokines which aggravates inflammation. In rats with CKD, curcumin treatment significantly decreases macrophage infiltration and curbs cytokine mediated increase of kidney phospholipase and cyclooxygenase which can lead to the formation of inflammatory eicosanoids and adversely affect kidney function [14]. In immune complex-mediated complement-dependent glomerulonephritis, another model for chronic renal failure, Jacob *et al.* showed that curcumin significantly improved renal function, reduced glomerulonephritis, decreased IgG deposits, and C9 deposits indicating reduced complement activation [48]. Furthermore, mRNA expression of proteins contributing to inflammation and fibrosis such as monocyte chemoattractant protein-1 and transforming growth factor-β, matrix proteins, fibronectin, laminin and collagen was thwarted by curcumin [48].

## 3. Major Pathologies Affecting CKD

### 3.1. Hypertension

Hypertension is the most common comorbidity in CKD, at least 85% of patients with stage 3 CKD or greater have hypertension [49]. According to the European Society of Cardiology and European Society of Hypertension, blood pressure targets for CKD patients should be less than 140/90 mm Hg for patients with non-proteinuric CKD, and less than 130/80 mm Hg for those with proteinuria [50]. In 5/6 nephrectomized model of CKD, Tapia *et al.* showed curcumin significantly reduced systolic BP within 15 days of curcumin therapy [51,52]. In our model of CKD we also found a significant drop in blood pressure with curcumin treatment but only after 8 weeks [35].

### 3.2. Proteinuria

Although proteinuria is a manifestation of CKD we are considering it separately because proteinuria may accelerate the progression of renal failure to end-stage kidney disease. This has received support from the results of increasing numbers of experimental and clinical studies. Noxious substances in the proteinuric ultrafiltrate promote inflammation and complement activation that lead to inflammatory cell (macrophage) infiltration in the interstitium of the kidney resulting in sustained fibrogenesis [35,43,53]. Macrophages are conspicuous in the interstitial inflammatory infiltrate and lead to the progression of renal injury to the extent that macrophage numbers in renal biopsy predict renal survival in patients with CKD [53]. In a rat CKD model we have shown that curcumin significantly reduces proteinuria, macrophage infiltration in the kidney and ameliorates CKD [35]. It is possible the reduction of proteinuria seen in our study was associated with the decreased macrophage migration. In another study Tapia *et al.* have also shown that curcumin treatment in rats, reduced proteinuria by approximately 75% and was accompanied with decrease in glomerular hypertension, hyperfiltration, glomerular sclerosis, interstitial fibrosis [51]. In a small randomized, double-blind and placebo-controlled study, Khajehdehi *et al.* gave patients with diabetic nephropathy, turmeric capsules (one capsule containing 500 mg turmeric, of which 22.1 mg was the active ingredient curcumin) three times daily for 2 months and observed a significant reduction proteinuria and TGFβ [29]. The same group also found that turmeric treatment significantly decreased proteinuria and systolic blood pressure in patients with renal failure associated with lupus nephritis [54].

### 3.3. Cardiovascular System

The accumulation of uremic toxins in advanced CKD adversely affects almost all organ systems though the most notable effect is seen on the cardiovascular system [55]. Uremic toxins contain oxidants, inflammatory cytokines and other molecules which not only affect CKD but also modulate cardiovascular disease such as atherosclerosis and heart failure, the two major diseases responsible for the mortality in CKD patients [2,56,57]. Indeed atherosclerotic plaques of patients with CKD have markedly higher lipid volume, lower fibrous volume [58] and are significantly unstable [59]. Accumulation of inflammatory cells is important for the genesis of atherosclerosis. HMG-CoA reductase inhibitors (statins) were shown to regulate the expression of chemokines and cytokines such as MCP-1 and IL-6 hence controlling the migration of leukocytes to sub-endothelial sites of inflammation, thereby favorably affecting atherosclerosis [60]. Curcumin is known to decrease atherosclerosis by lowering plasma and hepatic cholesterol and also by reducing HMG-CoA reductase. Its beneficial effect was equivalent to lovastatin [61]. It also ameliorates atherosclerosis by increasing the expression of ABCA1, LXR, SR-B1 and increasing cholesterol efflux [62]. Hasan *et al.* reported 500 mg/kg/curcumin diet effectively improved atherosclerosis by reducing fatty streak formation and suppressing aortic expression of IL-6 and blood levels of several inflammatory cytokines [63]. Recently we have shown that LDL receptor knockout mice on high fat cholesterol diet had significant atherosclerosis and glucose intolerance which was associated with increased inflammatory markers [64]. Daily administration of 100 mg/kg curcumin not only reduced the inflammatory markers but also improved atherosclerosis and glucose intolerance [64].

Heart failure commonly seen in patients CKD usually starts with cardiac hypertrophy. In CKD animals we have shown that curcumin attenuated cardiac hypertrophy and remodeling through deactivation of multiple hypertrophic signaling pathways [33]. Morimoto *et al.* have shown that curcumin prevents heart failure in hypertensive rats by inhibiting p300 histone acetyltransferase activity [65]. Non-hemodynamic mechanisms such as inflammation have been proposed to play a major role in the progression of CKD in the setting of congestive heart failure [66]. In addition, venous congestion associated with congestive heart failure may increase gut absorption of endotoxin leading to additional inflammatory responses, moreover, venous congestion itself is a stimulus for peripheral synthesis and release of inflammatory mediators [66]. Clinical evidence for this pro-inflammatory state comes from observations that patients with severe HF have markedly elevated levels of tumor necrosis factor TNF-α, up-regulation of soluble receptors for TNF, and a number of interleukins including IL-1β, and IL-6, as well as several cellular adhesion molecules [66,67] and curcumin, antagonizing the expression or action various cytokines and chemokines, can ameliorate heart failure.

### 3.4. Diabetes

Insulin resistance promotes proliferation of renal cells, increases various growth factors productions such as TGFβ, up-regulates the expression of receptors such as angiotensin II type 1 receptor in mesangial cells, and decreases endothelial production of nitric oxide [68]. Cumulatively these changes lead to the progression of diabetic nephropathy which frequently leads to CKD. The effect of curcumin on diabetes is discussed in the article “Curcumin and Diabetes” by C.S. Paulose published in this series.

## 4. Curcumin: Action in/on the Gut despite Low Bioavailability

**Figure 2:**
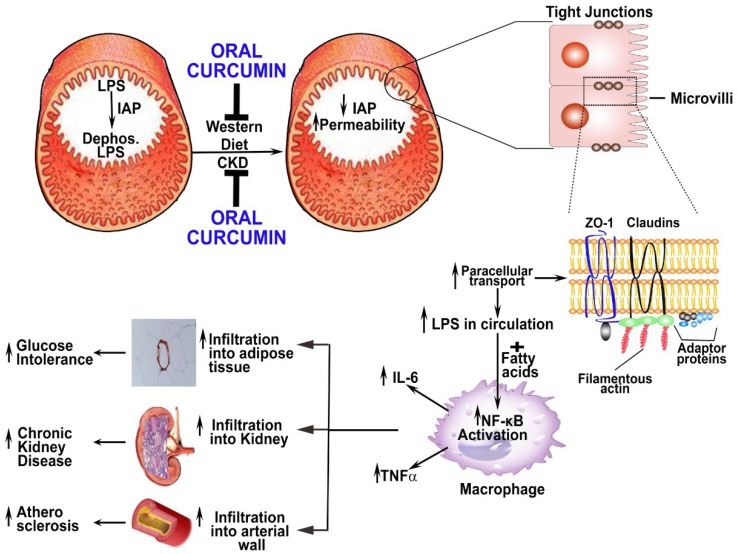
Proposed model for the role of intestinal barrier dysfunction in development of CKD, glucose intolerance and atherosclerosis and its modulation by curcumin. Intact intestinal barrier restricts the release of bacteria as well as bacterial products such as LPS into circulation. Consumption of Western diet as well as CKD affects this barrier function likely at multiple levels including decrease in Intestinal Alkaline Phosphatase (IAP) and/or increase in epithelial permeability resulting from a decrease in epithelial tight junction proteins (ZO-1, Claudins). IAP, an enzyme present in the lumen, detoxifies luminal LPS by catalyzing the removal of one of the two phosphate groups from the toxic lipid A moiety of LPS. Western diet-induced reduction in IAP activity, therefore, is likely to increase the levels of active LPS within the intestinal lumen. Luminal LPS-induced decrease in the expression of tight junction proteins ZO-1 and Claudin increases the paracellular transport of LPS into circulation; increased levels of circulating LPS are associated with CKD as well as Western-diet induced diseases such as atherosclerosis and Type 2 diabetes. Plasma LPS along with Western diet-induced increase in circulating fatty acids promotes macrophage activation and induce NF-κB-driven gene expression resulting in increased secretion of pro-inflammatory cytokines and chemokines. Activated macrophages also infiltrate into adipose tissue, kidney and/ or artery wall leading to the development of glucose intolerance, CKD and atherosclerosis, respectively. Oral administration of curcumin increases IAP activity as well as reverses the LPS-mediated decrease in tight junction proteins. Preservation of the intestinal barrier function at multiple levels (e.g., IAP, tight junction proteins) thus represents a novel mechanism by which poorly absorbed curcumin exerts potent anti-inflammatory effects leading to attenuation of CKD and/or Western diet-induced glucose intolerance and atherosclerosis.

High fat high cholesterol containing western diet is a major contributor to various disease process including atherosclerosis diabetes and CKD. We have shown that intestinal barrier is breached by Western diet [64]. Disruption of the intestinal barrier by Western diet leads to translocation of LPS from the gut to the circulation and increase in inflammatory cytokines [64]. Similarly uremic toxins associated with CKD which ranges from low molecular weight organic molecules such as indoxyl sulfate and p-cresyl sulfate [57] to high molecular inflammatory products such as LPS gets into the circulation leading to inflammation [69]. In addition, significant increase in plasma cytokines such as IL-1β, IL-6 and TNFα are also observed in CKD patients [2,57]. Uremic toxins can be classified according to the site of origin: (a) produced by endogenous metabolism; (b) microbial metabolism in the gut; and (c) ingested from exogenous sources [2]. Although majority of the toxins are produced by the first category a large number originate from intestinal microbial metabolism [2]. Therefore the intestine besides playing a role in atherosclerosis and diabetes also affects the pathophysiology of CKD. Indeed there are several articles showing that intestinal paracellular permeability and gut microbiota play a major role in development and prognosis of CKD [69,70,71]. We have reasons to hypothesize that curcumin can provide some of its beneficial effect by modulating intestinal permeability which will be another mechanism by which curcumin can abate renal failure. The bioavailability of curcumin in both man and animals is extremely poor [72,73,74]. However, in animal experiments when curcumin was compared with an existing therapeutic drug such as enalapril for CKD or lovastatin for atherosclerosis, it was found be of equal therapeutic efficacy [14,35,61]. Like curcumin both enalapril and lovastatin have active metabolites but neither drug has poor bioavailability like curcumin [75,76]. All things considered it can be speculated that curcumin might also act at the intestinal level. There is emerging evidence that intestinal microbiota is significantly altered in patients with CKD leading to increased permeability of bacterial metabolites and toxins [70,77]. Vaziri *et al.* have shown that there is a marked reduction of intestinal tight junction proteins claudin 1, occludin and ZO-1 in CKD rats and there was a close association between the renal function and breakdown of the intestinal barrier in these animals [70,78,79]. The disruption of intestinal barrier can lead to increased paracellular permeability of uremic toxins such as LPS and aggravate inflammation [64,69]. Sun *et al.* have shown LPS, a potent inflammatory mediator, enters the circulation of the CKD patients due to impaired intestinal barrier function and promote cardiovascular complications that often occur in patients with late-stage CKD [69]. We and others have shown that intestinal flora modulation by antibiotics, caused marked reductions in circulating LPS [64,80] this can result in decreased macrophage migration to tissues. Moreover, uremic toxins can enhance macrophage response to LPS [81] and LPS can increase the expression of pro-inflammatory cytokines [64,82,83]. We have shown that animals on high fat high cholesterol containing western diet had significantly high levels of non-absorbable dextran and LPS in the circulation suggesting a breakdown of the intestinal barrier, and this phenomenon was blocked by curcumin [64]. The increase in LPS was associated with significant increase in pro-inflammatory cytokines [64]. LPS mediated increase in pro-inflammatory cytokines such as TNFα and IL-1β can also aggravate the breakdown of the intestinal barrier by decreasing transcription of tight junction proteins and inducing cytoskeleton mediated redistribution of tight junction proteins [84]. We demonstrated that LPS mediated decrease in the expression of tight junction proteins (claudin and ZO-1) in Caco2 cells was abated by curcumin [64]. Furthermore, intestinal alkaline phosphatase (IAP) is a part of the intestinal luminal’s first line of defense and catalyzes the removal of one of the two phosphate groups from the toxic lipid A moiety of LPS producing monophosphoryl-LPS which renders LPS inactive [85]. Gastrointestinal administration of exogenous IAP ameliorates gut inflammation and favors gut tissue regeneration [86]. Recently we have shown that curcumin by increasing the catalytic activity of the intestinal alkaline phosphatase obliterates the breach in intestinal permeability and restores gut function [64]. Moreover, curcumin has significant antibacterial activity towards both Gram-positive bacteria and Gram-negative bacteria [87] which can modulate intestinal microbiota. It is clear that curcumin can modulate gut microbiota, prevent inflammation and preserve intestinal barrier function. Taken together, these effects of curcumin on the intestine may be another mechanism by which curcumin can provide beneficial effect in ameliorating inflammatory diseases such as CKD, atherosclerosis and diabetes. Figure 2 depicts the beneficial effects of curcumin on intestinal permeability and subsequent effects promoting the down-regulation of various inflammatory biomarkers and inhibition of macrophage infiltration culminating in the abatement of CKD and associated disorders.

### Bioavailability

Curcumin is a unique molecule which can prevent inflammation by acting at multiple sites. By preserving intestinal barrier function curcumin can prevent inflammation at the gut level and prevent systemic inflammation if adequate concentration is achieved in the circulation. Nanoparticle technology, liposomal encapsulation, polylactic-co-glycolic acid (PLGA) encapsulation, cyclodextrin, *etc.* have been used to improve the bioavailability of curcumin [88]. In a recent study it was shown that nano-emulsion curcumin given orally had a tenfold increase in the AUC and more than 40-fold increase in the Cmax compared with suspension curcumin in 1% methylcellulose given by the same route [89]. However, the bioavailability aspect of curcumin is discussed in more detail in the article by S. Shreeram “Bioavailability of Curcumin” published in this series. It can be speculated that the traditional curcumin which acts at the intestinal level and more bioavailable curcumin will become powerful and an innovative way to combat CKD and other inflammatory disorders.

## 5. Summary

In a recent Lancet editorial it was mentioned “During the past 10–15 years, several therapies, such as angiotensin-converting enzyme inhibitors or angiotensin II receptor antagonists, have delayed progression of kidney diseases. However, these achievements in renal medicine do not extend to all people. In low-income and middle-income countries, renal care is generally inadequate because of economic constraints and renal disease gets little attention from health policy makers [90]. Curcumin is a cheap nutritional ingredient with negligible side effect. It is also clear that curcumin has significant anti-inflammatory, anti-oxidant and various other features which make it a strong candidate to be included in therapeutic armamentarium for treating CKD. However, there are no big clinical trials on the horizon to test the efficacy of curcumin in CKD. Although, curcumin may produce its effect by modulating intestinal permeability, the efficacy of curcumin can be further enhanced if it can antagonize inflammatory mediators in the circulation. Unfortunately, poor bioavailability becomes the major obstacle for this compound. Clinical trials are expensive and very few would venture to try a compound with poor absorption. Fortunately, there are a large number of studies which are solely geared to improve the bioavailability of this compound. Furthermore, various chemical analogs of curcumin are showing promise. Hence, in the near future, it is quite likely a better absorbed curcumin or an analog along with traditional curcumin modulating intestinal permeability may find an application in the therapy of CKD.
